# Modeling HIV-1 Induced Neuroinflammation in Mice: Role of Platelets in Mediating Blood-Brain Barrier Dysfunction

**DOI:** 10.1371/journal.pone.0151702

**Published:** 2016-03-17

**Authors:** Letitia D. Jones, Joseph W. Jackson, Sanjay B. Maggirwar

**Affiliations:** Department of Microbiology and Immunology, University of Rochester School of Medicine and Dentistry, Rochester, New York, United States of America; Rutgers University, UNITED STATES

## Abstract

The number of HIV-1 positive individuals developing some form of HIV-associated neurocognitive disorder (HAND) is increasing. In these individuals, the integrity of the blood-brain barrier (BBB) is compromised due to an increase in exposure to pro-inflammatory mediators, viral proteins, and virus released from infected cells. It has been shown that soluble CD40L (sCD40L) is released upon platelet activation and is an important mediator of the pathogenesis of HAND but the underlying mechanisms are unclear, emphasizing the need of an effective animal model. Here, we have utilized a novel animal model in which wild-type (WT) mice were infected with EcoHIV; a derivative of HIV-1 that contains a substitution of envelope protein gp120 with that of gp80 derived from murine leukemia virus-1 (MuLV-1). As early as two-weeks post-infection, EcoHIV led to increased permeability of the BBB associated with decreased expression of tight junction protein claudin-5, in CD40L and platelet activation-dependent manner. Treatment with an antiplatelet drug, eptifibatide, in EcoHIV-infected mice normalized BBB function, sCD40L release and platelet activity, thus implicating platelet activation and platelet-derived CD40L in virally induced BBB dysfunction. Our results also validate and underscore the importance of EcoHIV infection mouse model as a tool to explore therapeutic targets for HAND.

## Introduction

Human immunodeficiency virus type-1 (HIV-1) currently infects approximately 36.9 million people worldwide and thus, is a global health concern [1]. The advent of combination antiretroviral therapy (cART) has played a pivotal role in reducing viral load and has dramatically decreased the death rate from HIV and acquired immune deficiency syndrome (AIDS) [2]. The central nervous system (CNS) is a major target for HIV, such that the virus enters the CNS early and remains for the duration of the infection [3]. Under normal physiology, the blood-brain barrier (BBB) serves to protect the CNS by separating it from peripheral blood [4]. BBB dysfunction, a hallmark of HIV-induced inflammatory response in the CNS, culminates in neurocognitive deficits ranging from mild to severe forms, known as HIV associated neurocognitive disorder (HAND), regardless of cART; 50% of HIV infected individuals are predicted to develop some form of HAND regardless of antiretroviral therapy [5]. This dysfunction is believed to be, in part, due to an increased expression of adhesion molecules on the surface of the endothelium, contributing to increased immune surveillance and loss of endothelial tight junction (TJ) protein levels following chronic exposure to the virus, viral proteins, and inflammatory mediators secreted by the infected and activated cells [6, 7]. Along with limiting paracellular flux, these junctions, composed of proteins such as claudin-5, occludin, etc, creates a seal amongst adjacent endothelial cells that selectively regulates access into the CNS and thus mediates transport of nutrients and other important components into the brain [8]. Disruption of TJs has been well described in HIV-1-infected patients and has been associated with an accumulation of HIV-1-infected macrophages in the brain [9]. When the BBB is compromised, permeability of the BBB is altered, resulting in increased leukocyte trafficking into the CNS, ultimately yielding a neurotoxic environment [7]. However, it is still unclear as to when the BBB dysfunction occurs during infection.

Understanding the pathological progression of HAND in humans is difficult due, in part, to analysis being largely limited to the availability of post mortem tissue samples. Even then, the collected data is a ‘snap shot’ of the disease terminally, and can be complicated by opportunistic infections and syndromes with disputed relations to HIV, thus creating a conundrum in being able to examine the brain during the pre-symptomatic stages of infection. Efforts to overcome this challenge have been successful by way of animal models. Rhesus macaques infected with simian immunodeficiency virus (SIV) is one of the earliest animal models effective towards the study of HIV-1 induced neuropathogenesis, specifically due to the ability to evaluate early post-infection CNS events [10]. The SIV-infected macaques display immunosuppression and CNS disorders pathologically and behaviorally similar to what has been reported in patients infected with HIV-1 [11]. Studies have shown that viral DNA is detected in brain tissues of infected animals as early as two-days post-infection [12]. Brain endothelial cells that were isolated from animals with terminal AIDS demonstrated that there was a decreased expression of the TJ protein zonula occludens-1 (ZO-1), as compared to uninfected animals [13]. In addition, an earlier study revealed a decrease in ZO-1 and occludin in basal ganglia tissue from SIV-infected macaques with encephalitis [14]. While these results correlate with what has been shown in post-mortem tissues from HIV-encephalitis (HIVE) patients [15], the caveats of these experiments are that SIV is more rapid in its progression [16] and brain samples were analyzed in animals at a more advanced stage of infection. Furthermore, the ability to analyze tissues from SIV-infected macaques in early and late stages of infection is hampered by the cost to maintain these animals.

Feline immunodeficiency virus (FIV) is another model used to explore the early events of HIV within the CNS [17]. Similar to SIV-infected Rhesus macaques, felines inoculated intravenously with FIV developed neuropathic disorders similar to individuals infected with HIV-1 [18]. Infected felines progressed through the serial stages of infection similar to HIV-1: initial flu-like symptoms proceeded by a lengthy asymptomatic period and finally, a terminal symptomatic phase [19, 20]. Histological studies that correlates with the aforementioned phases created a visual representation of the brain parenchyma and revealed that during the acute phase of infection, lymphocytes trafficked simultaneously through the blood-brain and blood-choroid plexus barriers. Research with both SIV and FIV infection models suggest that neural disease persists despite the viral load, and is more correlative with the number of immune cells that have invaded the CNS [21, 22]. However, the mechanisms affecting BBB function and trafficking into the CNS are not well understood in any of the current models of HIV-1 infection.

It is thought that HAND is more strongly correlated with activated monocytes/macrophages and neuronal dysfunction rather than the presence of infected cells in the CNS, CNS viral load or neuronal death [23–25]. Since acute exposure to viral proteins exert effects on the BBB [4, 26–28], we speculated that the BBB dysfunction occurs early on during infection. To test this possibility, we employed a mouse- tropic derivative of HIV to infect mice, termed EcoHIV. This chimeric virus contains gp80 envelope protein of Murine Leukemia Virus’s (MuLV) for attachment and gp41 envelope protein of HIV for fusion. Since the ecotropic envelope on EcoHIV does not infect human cells [29–31], EcoHIV is considered less hazardous than HIV for laboratory work. Earlier evidence indicates that EcoHIV mimics certain aspects of HAND [31–33]. Thus, this is a safer and more cost effective animal model of HIV infection that can be used to study the pathologies related to HIV infection, including HAND.

Several hypotheses have been raised in an effort to explain BBB disruption during HIV infection, with the establishment of a chronic inflammatory environment via the release of proinflammatory molecules and viral proteins being the most widely accepted theory [34, 35]. Viral proteins, such as transactivator of transcription (Tat) and gp120, are known to induce BBB permeability [36]. Our lab has previously demonstrated that the increased BBB permeability induced by Tat is dependent upon soluble CD40L (sCD40L), which is a host molecule predominately derived from activated platelets [37]. However, these findings were limited in assessing the effects of total viral infection with HIV, focusing solely on the effects of the viral proteins, and may not accurately represent what occurs during infection.

Taken together, in the current study we aimed to verify if viral infection with EcoHIV and platelet activation thereof mediates BBB dysfunction through utilization of a novel infection model. We show here that EcoHIV infection results in an increase in BBB permeability and a loss in TJ protein expression as compared to uninfected mice. We further demonstrate that the changes in BBB permeability due to EcoHIV-infection are dependent upon platelet activation and increased levels of sCD40L, as mice deficient in CD40L expression did not have BBB defects. To further explore the role of platelets in mediating the dysfunction of the BBB, we treated EcoHIV-infected mice with eptifibatide, an approved antiplatelet drug used extensively to treat patients with acute coronary syndrome (ACS), and BBB functions were measured. In infected mice treated with eptifibatide, we observed a reduction in BBB permeability as compared to infected mice treated with saline. Ultimately, this work demonstrates that the EcoHIV mouse infection model can be used to study the CNS related pathologies of HIV infection. Additionally, the results here implicate platelets as mediators of BBB integrity loss and stress the potential benefit of adjunctive antiplatelet therapy to the normalization of the BBB.

## Materials and Methods

### Ethics statement

In accordance with the Animal Welfare Act and the National Institute of Health (NIH) guidelines, and the University Committee on Animal Resources of the University of Rochester Medical Center, all experiments involving laboratory animals were conducted according to protocols previously approved by the aforementioned entities. The Association for the Assessment and Accreditation of Laboratory Animal Care International (AAALAC) fully accredited the facilities managed by the Vivarium and Division of Laboratory Animal Medicine of the School of Medicine and Dentistry and the facilities are in compliance with state law, federal stature, and NIH policy. All animals used in these studies, strains C57BL/6 and B6.129s2-CD40lgtm1mx/J, were purchased from The Jackson Laboratory, Bar Harbor, ME.

### Reagents

EcoHIV, constructed from pNL4-3 and pNCA-WT, previously characterized and tested [31], was kindly provided by Dr. David Volsky (Mount Sinai Hospital, New York, NY). Fluorescein sodium salt (sodium fluorescein, NaF) and polyethylenimine was purchased from Sigma-Aldrich (St. Louis, MO). Eptifibatide was purchased from BACHEM (Torrance, CA). Adenosine diphosphate (ADP) was purchased from Chrono-log (Havertown, PA).

### Virus preparation

Virus stocks were prepared by polyethylenimine (1mg/mL) transfection of plasmid DNA into 293T human embryonic kidney cells and titered for their p24 HIV core antigen by p24 ELISA (Advanced Bioscience Laboratories, Rockville, MD) according to the manufacturer’s protocol. Briefly, 7.5x10^5^ 293T cells/well were cultured in a 6 well-plate and transiently transfected with 4 μg of pNL4-3 DNA. 293T culture supernatants were harvested 48 h after transfection and concentrated by centrifuging at 22,000 x *g* for 2 h at 4°C. Supernatant was removed and the viral pellet was resuspended in 300μl of Dulbecco’s Modified Eagle Medium (DMEM) (Life Technologies, Grand Island, NY), aliquoted, and stored at -80°C.

### Assessment of HIV viral load

Plasma collected from EcoHIV infected mice at 1 month and 2 months post-infection was diluted 1:21 with fetal bovine serum and analyzed within 24 hrs using Roche COBAS Ampliprep COBAS Taqman HIV-1 version 2.0 test. Two regions of the genome, LTR and Gag, are measured with a detectable viral load range from 20–10,000,000 copies/mL. This test is FDA approved for establishing prognosis and for monitoring response to therapy in patients known to have HIV-1 infection but is not FDA approved for the diagnosis of HIV-1 infection.

### Sodium fluorescein assay

Eight-week old male WT or male CD40L-deficient (homozygous; C57BL/6 background; CD40L KO) mice were i.p. injected with the fluorescent tracer sodium fluorescein (10 mg/mL in 100 μL PBS) for 1 hr. Mice were then anesthetized, and whole blood was collected via cardiac exsanguination. Platelet poor plasma (PPP) was then obtained following sequential centrifugation. Following blood collection, animals were perfused with 20 mL cold PBS through the left ventricle to remove any residual dye from the vasculature. Tissues were harvested and subsequently homogenized in cold PBS (1:10 weight per volume), subjected to precipitation in 15% trichloroacetic acid, and pH was adjusted using NaOH. Fluorescence in prepared tissues or plasma was read using a SpectraMax M3 Multimode Microplate Reader (Molecular Devices, Sunnyvale, CA) with excitation at 485 nm and emission at 530 nm. Permeability was determined as the ratio of brain fluorescence/plasma fluorescence for each animal and samples were analyzed as fold change compared to saline treated WT animals.

### ELISA

Eight-week old wild-type C57BL/6 (WT) male mice were given intraperitoneal (i.p.) injections of either saline (American Regent, Shirley, NY) or EcoHIV (1.0x10^5^ pg p24). At 1–4 and 8 weeks post-infection, blood was collected via cardiac exsanguinations. Whole blood was sequentially centrifuged and PPP was collected. p24 concentrations were measured in PPP samples from 3 weeks and 8 weeks post-infected mice using a p24 ELISA kit (R&D systems, Minneapolis, MN). sCD40L and platelet factor 4 (PF4) concentrations were measured in PPP samples from 1, 2, 4 and 8 weeks post-infection using a mouse sCD40L ELISA kit or PF4 ELISA kit (both supplied from R & D systems, Minneapolis, MN), according to the manufacturer’s protocol. S100 Calcium Binding Protein B concentrations were measured in PPP samples from 4 and 8 weeks post-infection using a mouse s100B ELISA kit (Antibodies-Online, Inc., Atlanta, GA).

### Tail bleeding assay

Tail bleeding times were determined as described previously [37]. In brief, eight-week old WT mice were infected with EcoHIV (1.0x10^5^ pg p24) and treated with saline or eptifibatide (10 μg/mouse). At the indicated time points, mice were anesthetized and a 2 mm cut was made in the tip of the tail. The bleeding time was recorded from the time bleeding started until it stopped completely.

### Flow cytometry

Mouse whole blood was treated with either 10 μM ADP or 1.0x10^5^ pg p24 EcoHIV for 30 min, 1 hr, and 2 hrs at 37°C. Subsequently, blood was processed and analyzed for platelet activation by flow cytometry. Briefly, whole blood was fixed with 4% paraformaldehyde and washed twice with 1 ml staining buffer (1x PBS containing 2% BSA). Red blood cells (RBCs) were then lysed using ACK lysis buffer (Life Technologies, Grand Island, NY) and the remaining cells were washed twice in staining buffer. Cells were stained with 2.5 μl anti-mouse CD61-PE (AbD Serotec, Oxford, U.K.) and 1 μl anti-mouse CD62P-FITC (BD Biosciences, San Jose, CA). Following the staining, samples were acquired using a flow cytometer (Accuri C6; Accuri Cytometers, Ann Arbor, MI). Platelets were gated based on forward and side scatter, followed by assessment of the platelet activation marker CD62P. Sizing beads (Mega Mix; BioCytex, Marseille, France) were used to delineate the platelet gate (0.9–3 μm). Unstained cells and cells stained with only CD61 were used as controls. 20,000 events were acquired to measure platelet percentages and activation in terms of CD62P expression.

### Immunoblot analysis

Homogenized brain tissue lysates were prepared in ELB buffer (50 mM HEPES (pH 7), 250 mM NaCl, 0.1% Nonidet P-40, 5 mM EDTA, 10 mM NaF, 0.1 mM Na3VO4, 50 μM ZnCl2, supplemented with 0.1 mM PMSF, 1 mM DTT, and a mixture of protease and phosphatase inhibitors) and cellular debris was removed by high-speed centrifugation. The supernatants were collected and protein concentrations were determined using the Bradford protein assay. Samples (34 μg protein/lane) were separated on SDS-polyacrylamide gel and electrophoretically transferred to Hybond ECL nitrocellulose membrane (GE Healthcare Bio-Sciences Corporation, Piscataway, NJ). The membranes were then analyzed for immunoreactivity for antibodies against: claudin-5, occludin, ZO-1, I-CAM-1 and α-tubulin (Santa Cruz Biotechnology, Dallas, TX). Bound antibodies were detected using species-specific infrared-conjugated secondary antibodies (Li-Cor Biosciences, Lincoln, NE), followed by visualization using a Li-Cor Odyssey Infrared Imaging System (Li-Cor Biosciences, Lincoln, NE). Densitometry was performed on the resulting blots using Image Studio Lite (Li-Cor Biosciences, Lincoln, NE).

### Immunohistochemistry

Eight-week old wild-type C57BL/6 (WT) male mice were given intraperitoneal (i.p.) injections of either saline or EcoHIV (1.0x10^5^ pg p24). At 8 weeks post-infection, mice were anesthetized via an i.p. injection of a ketamine (100 mg/kg) and xylazine (10 mg/kg) cocktail. Subsequently, brains from dissected mice were sectioned in the sagittal plane, fixed in 4% paraformaldehyde and embedded in paraffin blocks. Immunohistochemistry was performed using standard methodology. Briefly, serial sections (5 μm in thickness) were baked at 65°C for 20 min followed by deparaffinization and rehydration. The tissue was then subjected to antigen retrieval by incubating the sections at 100°C in 10 mM sodium citrate buffer (pH 6.0) for 20 min. To block nonspecific antibody binding, the slides were incubated with 1% goat serum in 1x PBS and 0.1% Triton X-100 (Sigma-Aldrich, St. Louis, MO). For immunolabeling, a rabbit polyclonal antibody directed against claudin-5 (1:100 dilution in 5% donkey serum; Invitrogen, Carlsbad, CA) was used. Tissue sections were then rinsed, and secondary antibody, donkey anti-mouse conjugated to Alexa Fluor 594 (1:200 dilution in 5% donkey serum; Invitrogen, Carlsbad, CA), was added and incubated for 1 hr. The slides were subsequently mounted with ProLong antifade reagent containing DAPI (Life Technologies, Carlsbad, CA). Images were acquired using a Ziess Axiovert 200 Fluorescent Microscope purchased from Ziess (Thornwood, NY) and analyzed with the software Image-Pro Plus (MediaCybernetics, Rockville, MD).

### Quantitative reverse transcription polymerase chain reaction

Changes in the expression of tight junction genes from mouse brain samples were analyzed by quantitative RT-PCR (qRT-PCR). Total RNA was isolated using RNeasy Mini Kit (Qiagen, Valencia, CA), according to the manufacturer’s protocol. First strand complementary DNA synthesis was then performed using the Bio-Rad iScript cDNA Synthesis kit (Bio-Rad, Hercules, CA) and RT-PCR was completed using the Platinum Pfx DNA polymerase (Invitrogen, Carlsbad, CA). The following primers were used: Claudin-5 (forward) 5’-CCTTCCTGGACCACAACATC-3’ and (reverse) 5’-CGCCAGCACAGATTCATACA-3’; ZO-1 (forward) 5’—CGAGACACGGAGTTATAG and (reverse) 5’- GAAGGAAGGTGT GTAGAGRT-3’; Occludin (forward) 5’-GAGCTTACAGGCAGAACTAGAC-3’ and (reverse) 5’-CAGCCATGTACTCTTCACTCTC-3’; I-CAM-1 (forward) 5’- CACCGTGTATTCGTTTC-3’ and (reverse) 5’- GTCTGCAGG TCATCTTA-3’; V-CAM-1 (forward) 5’- CCAGATAGACAGCCCACTAAAC -3’ and (reverse) 5’- TCTCTCTCTCTCTCTCTCTCTCT -3’; GAPDH (forward) 5’-TGATGACATCAAGAAGGTGGTGAA-3’ and (reverse) 5’-TCCTTGGAGGCCATFTAGGCCAT-3’. qRT-PCR was performed on ABI Prism 7700 Sequence Detector (Applied Biosystems, Foster City, California) using a standard cycling protocol. Cycle threshold (Ct) values were determined and mRNA expression levels were calculated using 2−ΔΔCT method with expression level of claudin-5 mRNA normalized to GAPDH and represented as fold change.

### Complete blood counts

Complete blood counts were performed by collecting ~20 μl blood via retro-orbital sinus bleeds into glass capillary tubes coated with EDTA (Fisher Scientific, Pittsburgh, PA). Counts were performed using a Heska CBC-Diff Veterinary Analyzer (Heska, Fort Colllins, CO).

### Statistical analysis

For each experiment, statistical significance was determined using one-way ANOVA followed by Bonferroni’s test for multiple comparisons or unpaired t-test. Data from each replicated experiment is represented as mean ± SEM for each group with statistical significance indicated in each figure as *p<0.05, **p<0.01, ***p<0.001, ****p<0.0001.

## Results

### EcoHIV infection increases blood-brain barrier permeability in mice

We have previously demonstrated that acute exposure to HIV Tat induces BBB permeability in mice [37]. We wished to examine this phenomenon further by utilizing a mouse model of HIV infection (EcoHIV infection).

**Fig 1:**
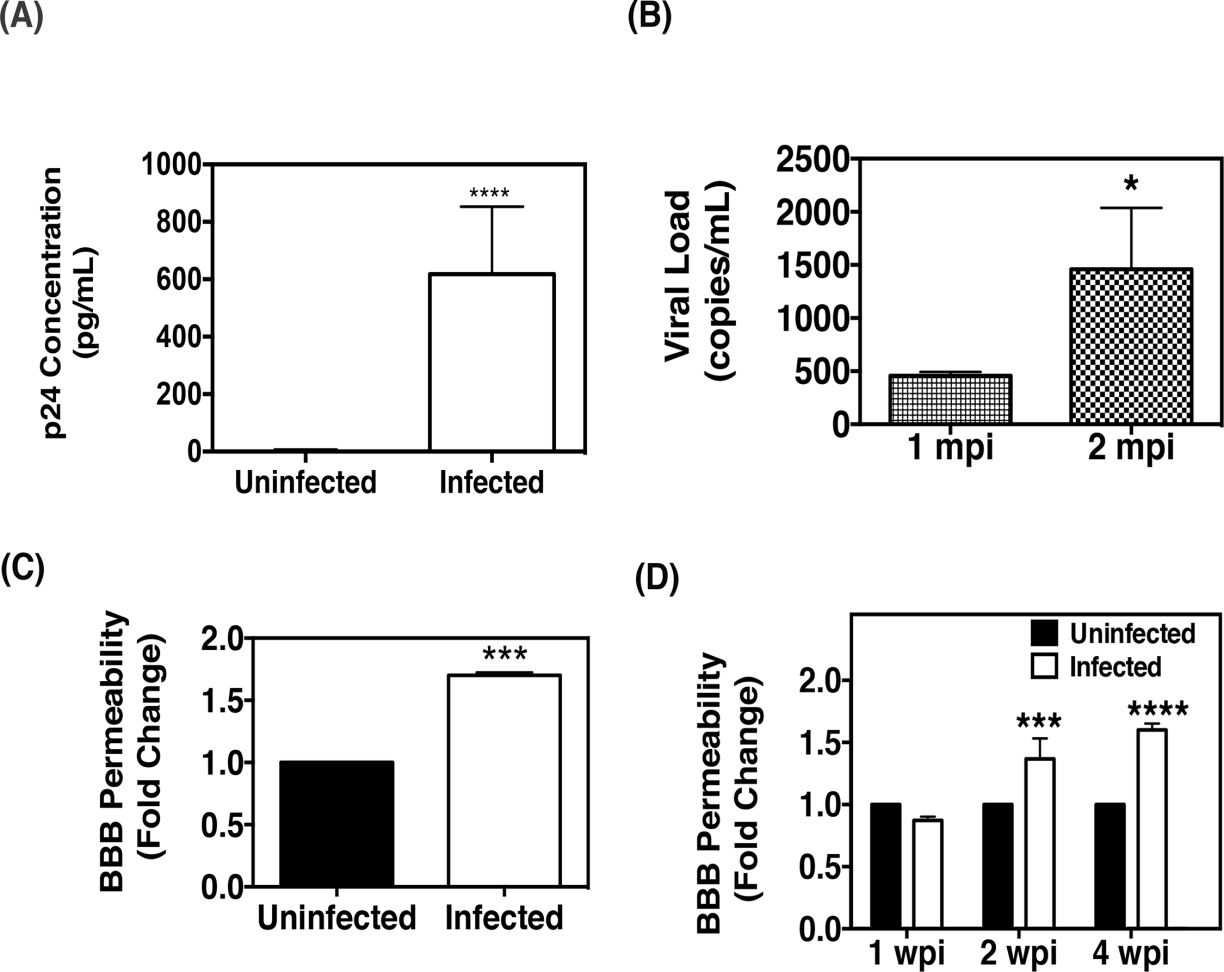
EcoHIV infection induces BBB permeability in mice. **(A)** Plasma concentrations of p24 in wild-type C57BL/6 (WT) mice were measured via ELISA three weeks post-infection (uninfected: n = 6, infected: n = 6). **(B)** EcoHIV viral load in plasma from WT mice was quantitated 1 month and 2 months post-infection (mpi) by PCR and the results were shown as the number of viral copies/mL (n = 6/time point). **(C)** WT mice were infected with EcoHIV for two months, and the fluorescent tracer, sodium fluorescein (NaF), was then used to assess BBB permeability. EcoHIV significantly increased BBB permeability as compared to uninfected animals (uninfected: n = 5; infected: n = 6). The values are presented as fold increase in the ratio of brain versus plasma concentrations of NaF. (**D)** WT mice were left uninfected or infected with EcoHIV (uninfected and infected: n = 4/time point). BBB permeability was analyzed at the indicated time points (weeks post infection, wpi) via NaF exclusion.

We first verified the extent of EcoHIV replication in wild-type C57BL/6 (WT) mice. Nine-week old mice were injected with either saline or EcoHIV intraperitoneally and subsequently sacrificed at 3 weeks post-infection. Blood was then collected via cardiac exsanguination and the plasma from these mice was assessed for levels of viral capsid protein, p24, by ELISA. As shown in Fig 1A, significantly higher levels of p24 were detected in EcoHIV-infected mice as compared to uninfected mice, verifying productive viral replication. We further confirmed viral replication by measuring viral RNA copies in the plasma of mice infected for either 1 month or 2 months using Cobas Ampliprep. We observed measureable viral load by 1 month post infection, which was significantly increased by 2 months post infection, indicating active viral replication (Fig 1B). Collectively, our data demonstrates that EcoHIV successfully replicates in mice and is consistent with studies performed by others [31–33, 38].

In an effort to explore the effect of EcoHIV infection on BBB function, the sodium fluorescein (NaF) assay was employed. Sodium fluorescein, a 376 dalton fluorescent dye, is commonly used as a marker of BBB permeability because it can only cross the barrier paracellularly once the BBB has been compromised [39–41]. In order to assess the degree of BBB permeability, the ratio of brain fluorescence to plasma fluorescence for each animal is determined. We demonstrate that at 2 months post-infection, BBB permeability was significantly increased in EcoHIV-infected mice as compared to uninfected mice (Fig 1C). Moreover, we established that the BBB is compromised as early as 2 weeks post-infection, suggesting that BBB dysfunction occurs early during infection in this model (Fig 1D). While sodium fluorescein assays are used frequently for measuring BBB permeability, it is not a realistic examination in clinical settings. Therefore, measuring the plasma concentration of calcium-binding protein B (S100B), a protein predominately secrected by astrocytes and used to clinically measure BBB damage in traumatic brain injury patients [42], substantiated BBB permeability analysis in EcoHIV-infected mice. We show in EcoHIV infected mice two-months post-infection, concentrations of S100B was significantly higher as compared to uninfected control mice; thus confirming our previous analysis of BBB permeability via sodium fluorescein assay (S1A Fig).

Collectively, this data demonstrates that successful replication of EcoHIV in mice results in increased BBB permeability early in the course of infection, highlighting the potential of this model in examining the mechanisms involved in the CNS related pathologies of HIV infection.

### EcoHIV induces platelet activation

Atypical platelet activation is increasingly identified as a main contributor in inflammatory disorders such as atherosclerosis, asthma and inflammatory bowel disorders [43–46]. We and others have reported that platelets circulate in a more activated state within HIV-1 infected individuals [47–49]. Furthermore, we have demonstrated that sCD40L, which is predominately released from platelets [50], is increased in the plasma and CSF of HIV-infected patients with HAND as compared to infected, non-cognitively impaired individuals [51]. Thus, we aimed to assess the role of EcoHIV infection on platelet activation.

**Fig 2:**
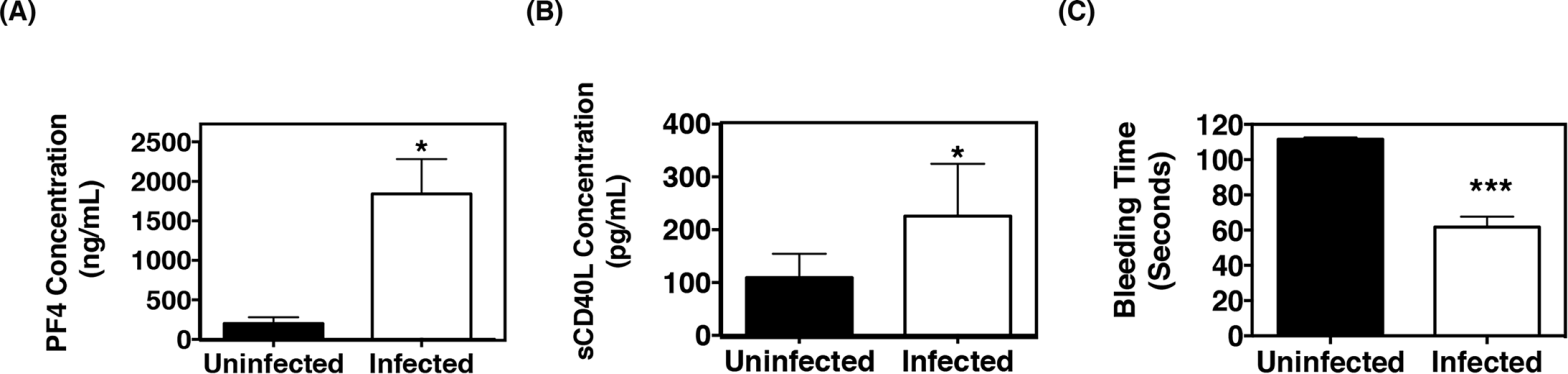
EcoHIV infection activates platelets *in vivo*. (**A**) Plasma concentrations of platelet factor 4 (PF4) and **(B)** soluble CD40L (sCD40L) in wild-type C57B/L 6 (WT) mice that were uninfected (n = 9) or infected with EcoHIV (n = 11) were measured via ELISA. Plasma levels for each of these platelet-derived mediators were significantly increased, indicating EcoHIV activates platelets *in vivo*. (**C**) Bleeding time in mice infected with EcoHIV (n = 11), decreased significantly as compared to uninfected mice (n = 9), indicating increased platelet activation.

WT mice were infected with EcoHIV and subsequently sacrificed 2 months post-infection. Plasma was collected and analyzed for the platelet releasates, platelet factor 4 (PF4) and sCD40L. We demonstrate here that PF4 and sCD40L levels were significantly higher in infected mice as compared to uninfected controls, suggesting elevated activation of platelets in these mice (Fig 2A and 2B). In addition, the amount of time needed for the clotting cascade to induce a thrombus was reduced in infected mice as compared to uninfected mice, as determined using the tail bleed assay, indicative of increased platelet activation (Fig 2C).

**Fig 3:**
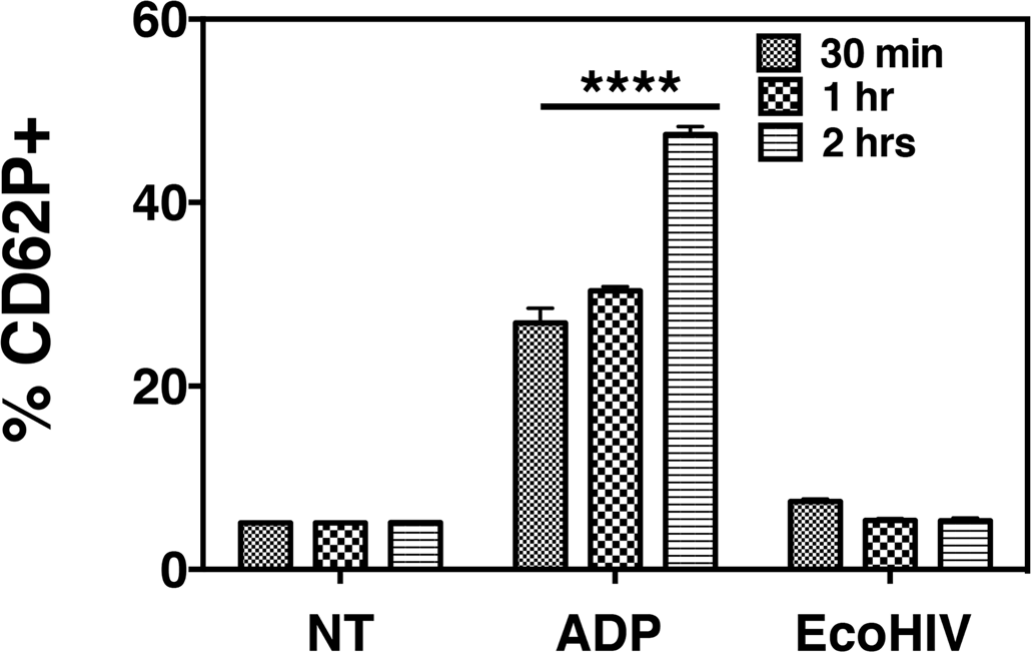
EcoHIV infection activates platelets indirectly. Whole blood was collected from wild-type C57BL/6 J (WT) mice (n = 3) and treated with 10 μM ADP or EcoHIV (1.0x10^5^ pg p24) for the indicated time periods (each group n = 3) *in vitro*. Activated platelets (CD61+ CD62P+) were then quantified by flow cytometry. The percentage of CD62P+ platelets in EcoHIV treated blood was comparable to non-treated (NT) blood, indicating the inability of EcoHIV in directly activating platelets.

We then wanted to determine if the observed platelet activation in infected mice was due to a direct effect of EcoHIV exposure. To test this notion, whole blood was collected from uninfected WT mice and treated with 10 μM ADP, a known platelet activator, or EcoHIV at 37°C for 30 minutes, 1 hr, and 2 hrs. Platelet activation was then analyzed by measuring surface expression of CD62P (P -selectin) via flow cytometry; CD62P is stored within the alpha granules of platelets that, upon activation, is subsequently transferred to the cell surface and is thus a commonly used marker for activated platelets. In contrast to treatment with ADP, EcoHIV did not induce platelet activation at any of the indicated time points (Fig 3). Consistently, our lab recently reported that whole blood collected from HIV negative donors left untreated or treated with HIV NL4-3, a human X tropic (X4) virus, for 1 hr also did not activate platelets as there was no significant difference in CD62P expression [52].

Taken together, this data suggests that EcoHIV infection indirectly induces platelet activation resulting in increased levels of platelet-derived soluble factors such as sCD40L and PF4. Further, this is comparable to what is observed with exposure of human whole blood to HIV, which suggests that the elevated platelet activation that occurs during HIV infection is not through direct virus-platelet interaction, strengthening the validity of the EcoHIV infection model.

### EcoHIV alters expression of certain tight junction proteins and adhesion molecules

Tight junction (TJ) proteins play a critical role in maintaining the integrity of the BBB. These junctions limit paracellular flux and promote endothelial cell-endothelial cell interaction, thus forming a barrier that strictly regulates the transport of macromolecules and inflammatory cells into the brain. In addition, post-mortem tissues from individuals that succumbed to HIVE displayed decreased TJ expression resulting in the increased accumulation of virus and infected macrophages in the CNS [15].

**Fig 4:**
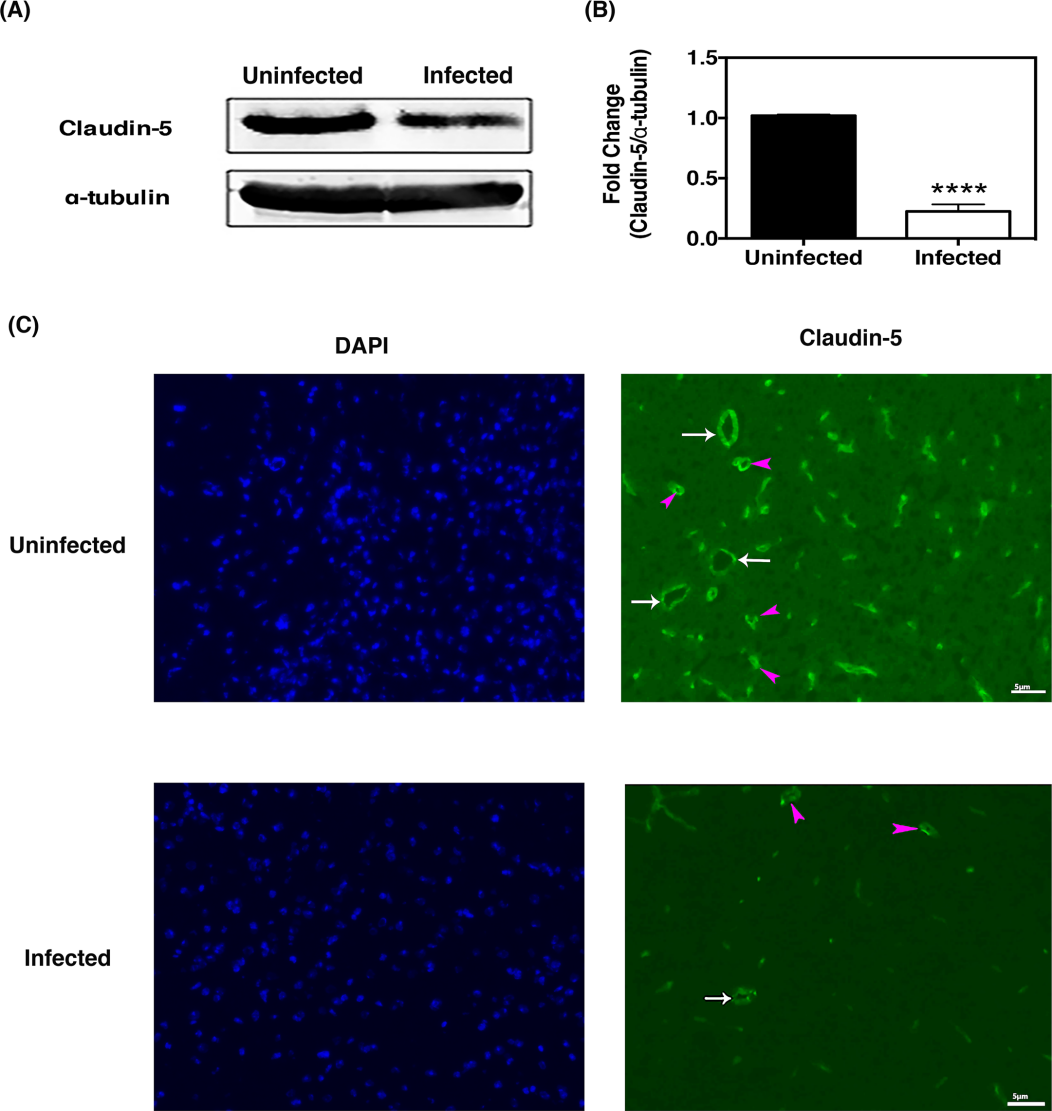
EcoHIV infection alters the expression of tight junction proteins. **(A)** Brain tissues from uninfected and EcoHIV-infected mice (two mpi) were homogenized, protein lysates collected and probed for claudin-5 expression via western blot. A representative blot image shows a decrease in claudin-5 expression in EcoHIV infected mouse. Corresponding densitometry quantification of claudin-5 protein levels from uninfected (n = 4) and EcoHIV infected (n = 6) mice confirms the decreased expression. **(B)** Brain tissues were sectioned in the sagittal plane and claudin-5 expression was assessed by immunohistochemistry. Claudin-5 (seen as green) expression was found to be relatively reduced in EcoHIV-infected mice; white arrows represent 5 μm vessels (scale marker), purple arrowheads represent microvessels smaller than 5 μm. Tissues were counterstained with DAPI (blue) for nuclei. The images presented here are representative of one experiment repeated twice.

Consistent with the observed changes in EcoHIV-induced BBB permeability, we aimed to determine if EcoHIV-infected mice demonstrated altered tight junction protein expression. At 2 months post-infection expression of claudin-5, a TJ protein known to be highly expressed in brain endothelium, was evaluated. We observed a marked loss of claudin-5 protein expression in infected mice compared to uninfected mice, as shown by immunoblot analysis (Fig 4A). The adjacent densitometry analysis was used to quantify the corresponding bands and confirmed decreased claudin-5 expression. HIV infection is often associated with a downregulation of TJ protein levels, which is thought to occur in brain endothelial cells of microvessels (i.e. capillaries) [53]. To test whether this is the case in EcoHIV-infected mice, we measured immunoreactivity of claudin-5 in brain tissues. As shown in Fig 4B, the brain microvessels (arbitrarily determined by the size <5 μM; denoted by purple arrows) showed a drop in claudin-5 levels following EcoHIV infection in mice. Interestingly, such a decrease in claudin-5 levels was also observed in macrovessels (> 5 μM; denoted by white arrows) of these mice. We further explored changes within the blood-brain barrier by analyzing the expression of additionial tight junction proteins ZO-1 and occludin, as well as junctional adhesion molecules, I-CAM-1 and V-CAM-1. Similarily to claudin-5, ZO-1 protein expression and transcription was decreased in EcoHIV infected mice whereas there was no change observed in occludin levels (S2A, S2B and S2E Fig). Further, we observed increased expression of I-CAM-1 and V-CAM-1 in EcoHIV infected mice (S2C and S2D Fig). Together, these results suggest that the increased BBB permeability observed in EcoHIV infected mice (Fig 1) is, in part, due to the increased endothelial activation as evidenced by increased I-CAM-1 and V-CAM-1 expression as well as the decreased expression of a key tight junction proteins, claudin-5 and ZO-1.

### EcoHIV-induced BBB permeability is dependent on CD40L

**Fig 5:**
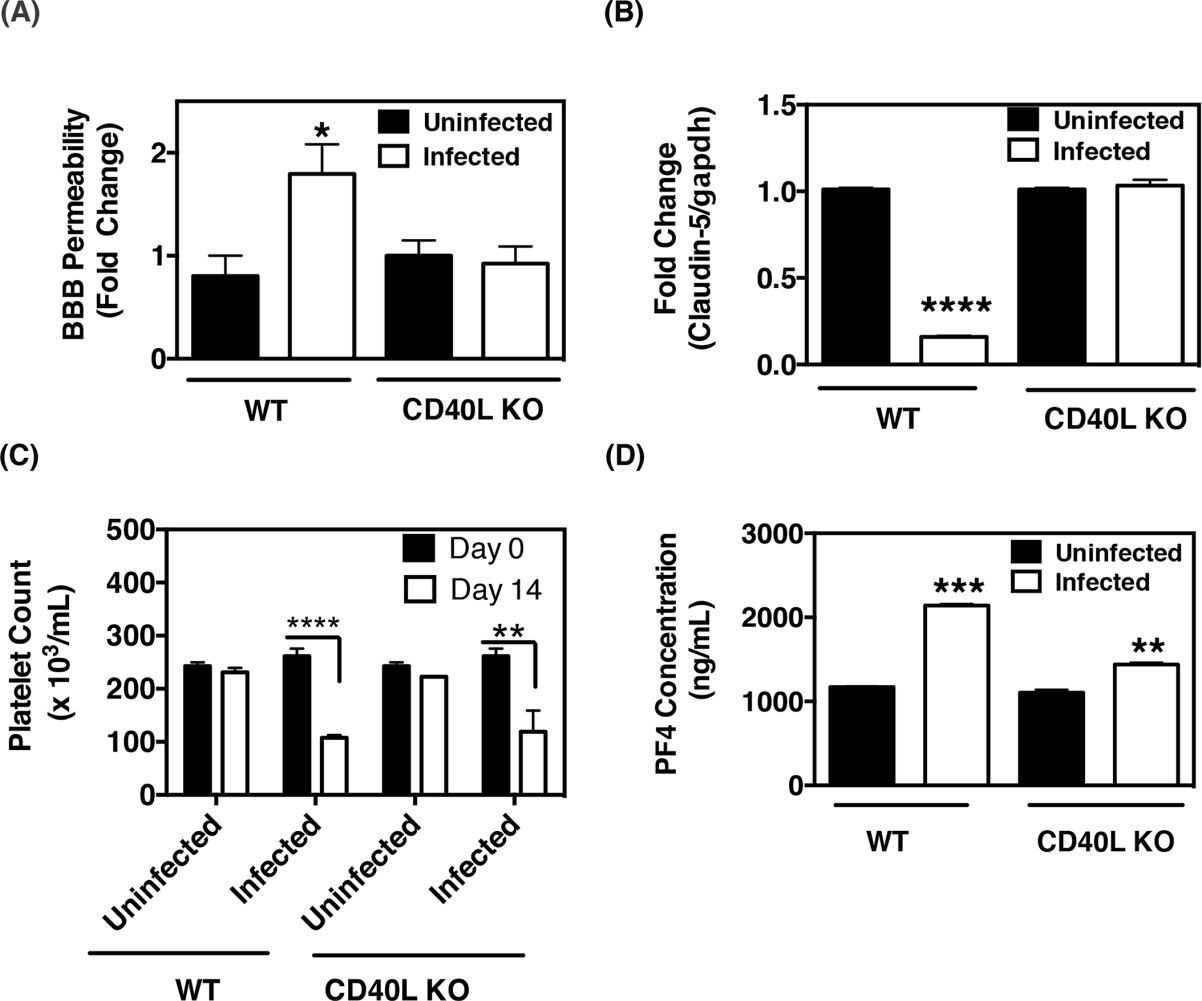
CD40L is required for EcoHIV-induced BBB permeability. **(A)** Wild-type (WT) or CD40L deficient (CD40L KO) C57BL/6J mice (both groups n = 3 each) were infected with EcoHIV, while control mice were left uninfected, for 14 days and sodium fluorescein (NaF) exclusion assay was performed to assess BBB permeability. The data reveals that the EcoHIV-induced increase in BBB permeability is dependent on normal expression of CD40L. The values are presented as fold increase in the ratio of brain versus plasma concentrations of NaF. **(B)** qRT-PCR analysis of claudin-5 expression in brain tissues harvested from WT and CD40L KO mice showed a reduction of claudin-5 transcripts in WT mice but not in the CD40L KO counterparts (both groups n = 3). **(C)** Complete blood counts revealed a reduction in platelet counts two wpi in both WT and CD40L animals (both groups n = 3). **(D)** Increased levels of PF4 were detected in the plasma of infected mice compared to their uninfected controls, suggesting a higher degree of platelet activation (WT and CD40L KO both n = 3).

We have previously reported that platelet activation induced by Tat yielded an increase in sCD40L that contributed to BBB permeability *in vivo* [37]. Hence, in an effort to explore the role of CD40L in EcoHIV-induced BBB permeability, WT and CD40L KO mice were infected with EcoHIV and subsequently sacrificed 2 weeks post-infection. BBB permeability was again assessed via NaF exclusion assay. It was found that EcoHIV-infection induced a significant increase in BBB permeability in WT mice at 2 weeks post-infection; however, this effect was suppressed in their CD40L KO counterparts (Fig 5A). Since we observed a decrease in claudin-5 expression in infected WT mice (Fig 4), and that BBB permeability was unchanged in infected CD40L KO mice (Fig 5A), we next analyzed claudin-5 transcript levels via qRT-PCR analysis to ascertain the role of CD40L on claudin-5 expression. After 2 weeks post infection, it was found that, compared to uninfected WT mice, claudin-5 expression was significantly lower in infected WT mice whereas infected CD40L KO mice expressed levels of claudin-5 comparable to the uninfected CD40L KO group (Fig 5B). Further, we report that infected CD40L KO mice expressed ZO-1 and I-CAM-1 at levels comparable to that of uninfected CD40L KO mice, which is in contrast to infected WT mice that had decreased ZO-1 and increased I-CAM-1 expression compared to uninfected WT counterparts (S3 Fig). Collectively, this data indicates that EcoHIV infection-induced BBB permeability and loss of claudin-5 expression is dependent upon CD40L.

As mentioned previously, sCD40L is predominately released from activated platelets, and it is thought that activated platelets are rapidly cleared from the circulation via immune cells, resulting in a decrease in platelet number. Further, it is established that HIV positive individuals display decreased platelets count (thrombocytopenia) post atypical platelet activation [54]. Hence, complete blood counts were performed in order to assess platelet number from both WT and CD40L KO mice. We observed that EcoHIV infection led to a significant reduction in platelet counts in both WT and CD40L KO mice that were infected for 2 weeks, as compared to uninfected controls (Fig 5C), indicating that platelets are activated in both groups of mice and subsequently cleared. In addition, PF4 levels were quantified and were found to be significantly increased in infected WT and CD40L KO mice compared to uninfected mice (Fig 5D).

Taken together, this data suggests that platelets are indeed activated and subsequently cleared in both WT and CD40L KO mice, which is similarly thought to occur in HIV-infected individuals, and that the EcoHIV-induced BBB permeability and loss of claudin-5 expression is dependent upon CD40L. Furthermore, other platelet derived pro-inflammatory releasates, such as PF4, are still present in the circulation of these animals, thus highlighting the importance of CD40L.

### Eptifibatide reduces BBB permeability

Since we have implicated platelet-derived sCD40L in the increased BBB permeability of EcoHIV infected mice, we sought to assess if antiplatelet therapy would alleviate this effect. Thus, we employed a clinically used antiplatelet drug, eptifibatide, which is an antagonist of the major mediator of platelet-platelet aggregation, integrin gpIIβ/IIIα (fibrinogen receptor), and is used to abrogate thromboembolisms and platelet activation in patients [55]. Previous reports have shown that gpIIβ/IIIα engagement upregulates CD40L, PF4 and CD62P surface expression [56, 57]. Hence, we first sought to verify the antiplatelet activity of eptifibatide in our mouse model.

**Fig 6:**
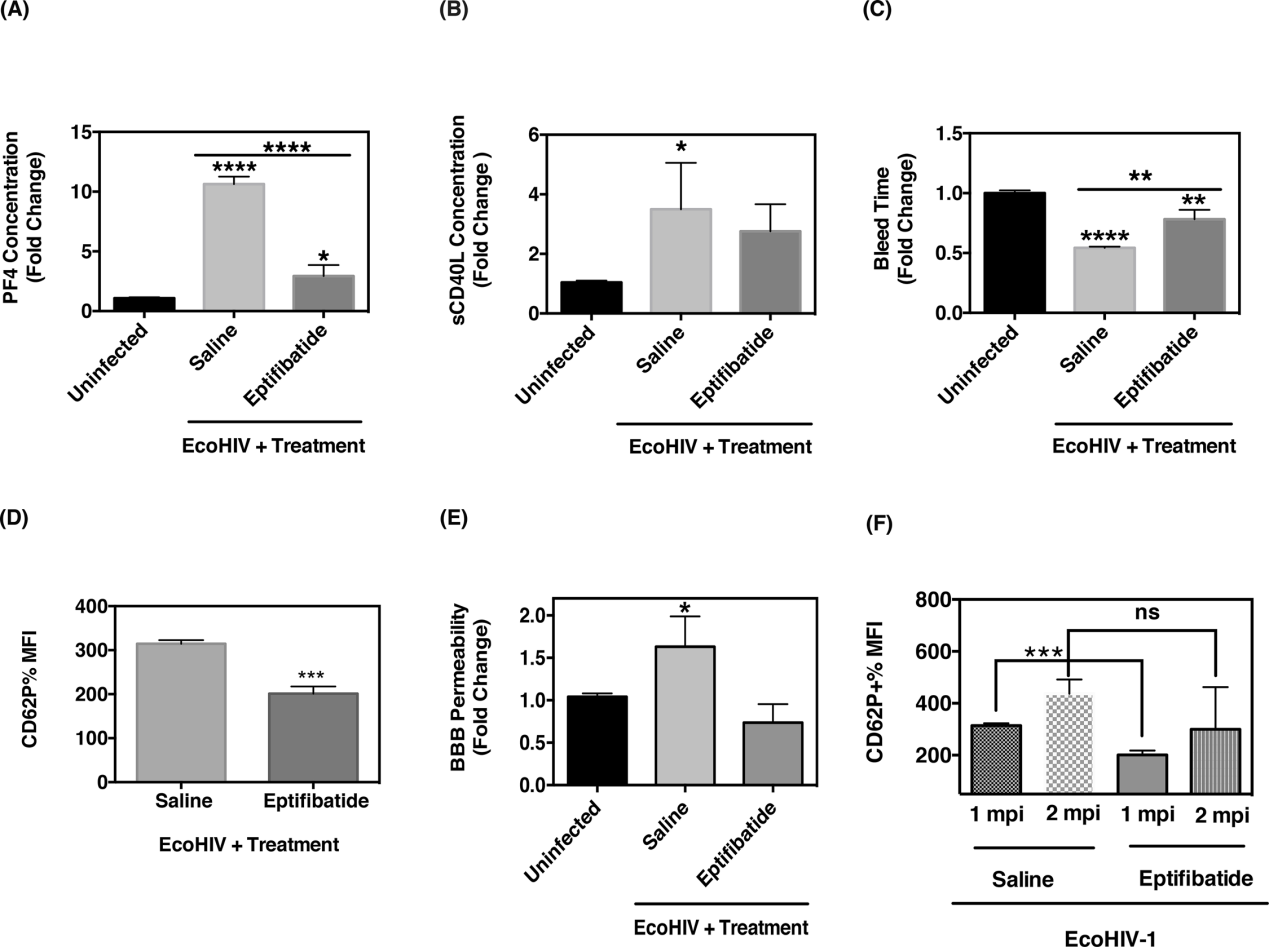
Antiplatelet treatment restores BBB function in EcoHIV-infected mice. Plasma concentrations of PF4 and sCD40L in EcoHIV infected mice were determined via ELISA following the treatment with either saline or eptifibatide (10 μg per mouse). As indicated, **(A)** PF4 (n = 6/group) and **(B)** sCD40L (n = 6/group) levels were found to be lower in eptifibatide-treated, infected mice compared to infected mice treated with saline. (**C**) Eptifibatide treatment of EcoHIV-infected mice (n = 6/group) increases the bleeding time. (**D**) Flow cytometry analysis of activated platelets (CD61^+^ CD62P^+)^ after one mpi with EcoHIV shows that mice receiving eptifibatide have significantly lower percentage of CD62P^+^ platelets than saline-treated control mice (n = 6/group). (**E**) Sodium fluorescein (NaF) exclusion assay revealed that eptifibatide treatment, with i.p. injections every 3 days over the course of 7 days, prevented EcoHIV-induced loss of BBB permeability one mpi (n = 6/group). **(F)** At two mpi CD62P^+^ expression rebounded in infected mice when eptifibatide treatment was ceased (n = 6/group).

WT mice were inoculated intraperitoneally with EcoHIV or left uninfected for one month. Subsequently, the indicated groups of mice received i.p. injections of either saline or 10 μg/200 μL of eptifibatide every 3 days over the course of 7 days. This dosage is comparable to the lowest clinical dose used in humans [58, 59]. Whole blood from infected mice treated with saline or eptifibatide were analyzed for platelet activation via measuring PF4 and sCD40L levels and compared to uninfected, untreated control mice. PF4 levels in infected mice treated with saline was approximately 2000 ng/mL± SEM, than infected mice treated with eptifibatide. Further, PF4 levels were significantly reduced in infected mice treated with eptifibatide as compared to saline-treated infected mice (Fig 6A). sCD40L levels were also found to be reduced in infected mice treated with eptifibatide as compared to infected mice treated with saline (Fig 6B). Furthermore, we also observed a significant increase in the time for bleeding following eptifibatide treatment, which was expected since eptifibatide inhibits platelet aggregation thus blocking the formation of a thrombus (Fig 6C).

Platelet activation was further assessed by measuring CD62P surface expression. Flow cytometry analysis confirmed that blocking gpIIβ/IIIα with eptifibatide in infected mice reduces CD62P expression as compared to infected mice treated with saline (Fig 6D). Next, we sought to ascertain the effect of eptifibatide treatment on EcoHIV-induced BBB permeability. Hence, we examined BBB permeability via NaF assay and discovered that, in contrast to infected mice treated with saline, permeability was restored to levels comparable to uninfected mice in eptifibatide-treated infected mice (Fig 6E). We also measured BBB permeability of infected mice treated with either saline or eptifibatide by measuring plasma levels of S100B via ELISA. We oberserved decreased plasma S100B levels in infected mice treated with eptifibatide as compared to saline treated mice (S1B Fig). Lastly, we ceased eptifibatide treatment for one month in a subset of infected mice, and subsequently, these mice were sacrificed at the timepoint of 2 months post-infection. Platelet activation was again measured by CD62P expression via flow cytometry. We observed that ceasing eptifibatide treatment in infected mice resulted in comparable CD62P expression to that of infected mice that were previously treated with saline (Fig 6F), suggesting a transient effect of eptifibatide in these mice. Taken together, these results suggests that continous antiplatelet therapy, such as eptifibatide, successfully reduces aberrant platelet activation and platelet-dependent BBB permeability increase that we observed in EcoHIV infected animals and would serve as a useful strategy in combatting HIV-induced BBB damage [60].

**Fig 7:**
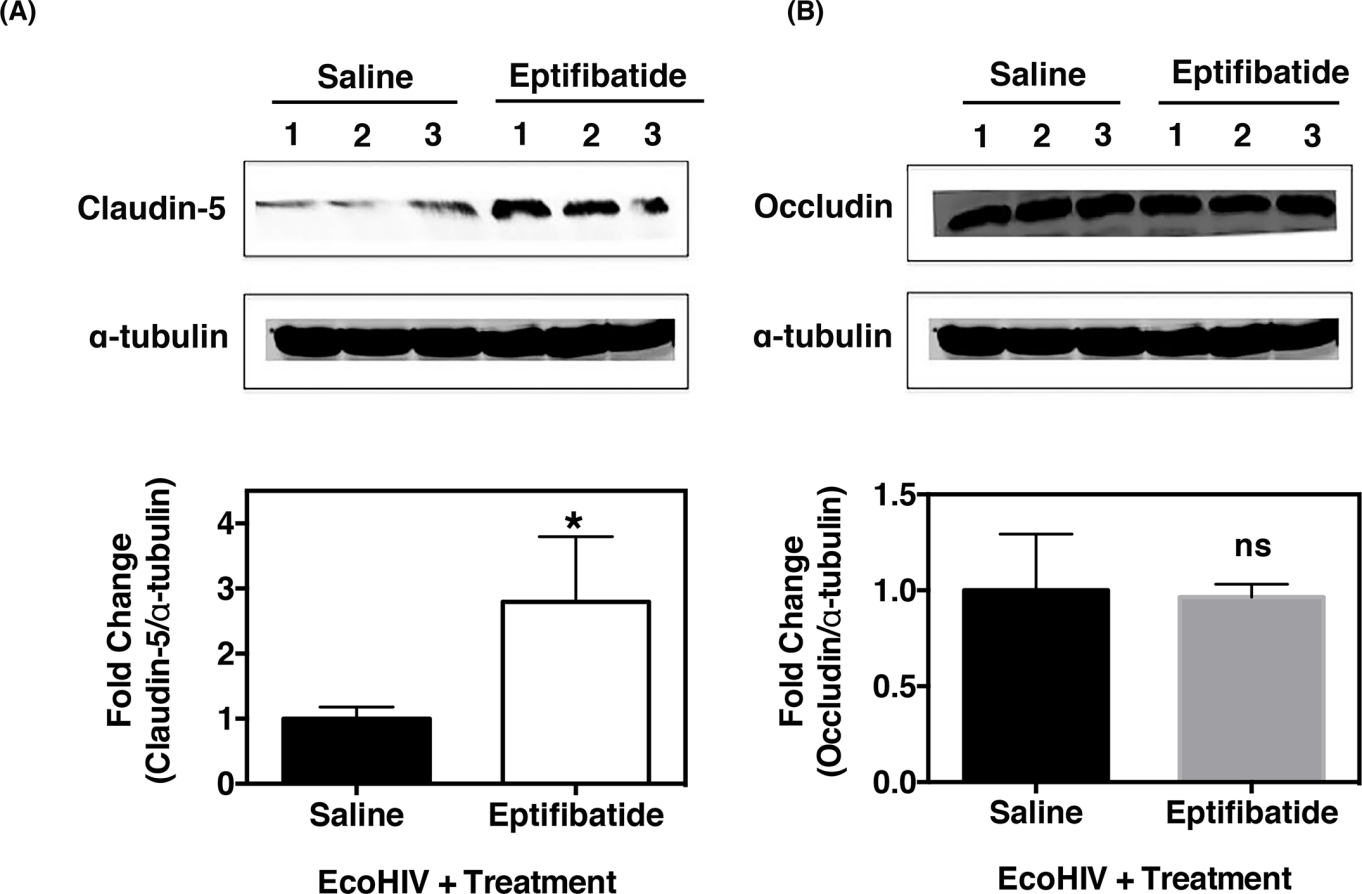
Eptifibatide treatment restores claudin-5 expression in EcoHIV-infected mice. **(A)** Expression of claudin-5 (n = 3) and **(B)** occludin (n = 3) proteins in brain tissues of EcoHIV infected mice was analyzed by western blot. One mpi, mice treated with eptifibatide showed higher level of claudin-5 expression compared to saline treated mice, whereas, occludin levels were not significantly different between the groups. The corresponding densitometric quantitation is shown below the respective blots.

Since we observed normalization of BBB permeability in infected animals treated with eptifibatide (Fig 6), we next sought to assess if eptifibatide treatment could thus restore BBB integrity through modulation of tight junction and adhesion protein levels. Thus, brain tissues from infected mice treated with either saline or eptifibatide were homogenized and analyzed via western blot. As demonstrated in Fig 7A, brain tissues from 1 month post-infected mice treated with eptifibatide had a two-fold increase in claudin-5 expression as compared to saline treated, EcoHIV-infected mice. This change was visually evident by the western blot but also by densitometry analysis, which confirmed an increase. However, occludin expression was not significantly different between saline and eptifibatide treated, EcoHIV-infected mice, one month post-infection (Fig 7B). Moreover, ZO-1 levels were increased and I-CAM-1 levels were decreased in EcoHIV-infected mice treated with eptifibatide as compared to that of EcoHIV-infected mice treated with saline (S4 Fig). Collectively, this data demonstrates that antiplatelet treatment, such as eptifibatide, successfully normalizes BBB permeability through modulation of the TJ proteins, claudin-5 and ZO-1, and reduces cellular adhesion molecule expression, ultimately strengthening the significance of antiplatelet therapy in reducing HIV-induced BBB dysfunction.

## Discussion

Antiretroviral therapies have proven to be effective in controlling viral replication, increasing the lifespan of HIV-1 positive individuals [2]. However, with HIV-1 infected individuals living longer, we have observed an increased prevalence in secondary illnesses associated with HIV-1 infection, such as HAND [2]. With >50% of all HIV-1 positive individuals estimated to develop some form of neurological disorder despite cART, there is a significant need for additional treatment(s) [5]. It is thought that HAND develops due to infected leukocytes, virus, and viral proteins trafficking through a compromised BBB, and establishing a neuroinflammatory environment in the CNS. Understanding the mechanisms of BBB dysfunction is thus a critical step in order to develop treatment for these patients, as currently there are no effective therapies.

Several animal models have been developed in an effort to understand the underlying processes to the pathogenesis of HAND, with posing challenges. Rhesus macaques infected with SIV displayed neuropathogenesis similar to humans and revealed that the virus infects the brain very early during infection as well as result in a decrease in tight junction protein expression [11–14]. Due to the inability to examine brain tissues of persons with HIV-1 early in infection, this model provided critical information [10, 12, 15]. However, a drawback with this model is that the viral infection progressed rapidly and sampled tissues were collected at more advanced stages of infection [16]. Furthermore, it is more costly to maintain these animals for prolonged periods of time. Another model used to study HAND is the FIV infection model, in which felines infected with FIV also displayed neuropathies similar to humans [18]. It was observed that infected leukocytes trafficked across the blood-brain and blood-choroid plexus barriers simultaneously. While both SIV and FIV models were effective in shedding light onto neurocognitive disorders, the mechanisms are not yet well understood thus leaving a void on how to abrogate the pathogenesis of HAND. Thus, due to ease of use and well-established methods to manipulate their genome, mice are considered valuable models for the research and development of vaccine therapies as well as to gather a basic understanding of HAND [61]; thereby helping to fill in that void. HIV is not infectious to rodents, therefore, creating a murine model to mimic human pathology is challenging. To overcome that challenge, transgenic mice expressing the proteins that are necessary for HIV replication were generated. An early study reported that transgenic mice expressing gp120 in astrocytes, exhibited significantly increased BBB permeability[62] and shortly after, another group reported impaired BBB permeability in transgenic mice expressing Tat in astrocytes [63]. Lastly, in a recent report, in a transgenic mice expressing full length HIV-1 provirus cloned from the lymphocytes of an HIV-1-infected patient, HIV infection systemically increased lipopolysaccharide (LPS) levels, that yields the BBB to be more susceptible to disruption by LPS, thereby increasing the ability of monocytes to enter the brain [64]. These transgenic mice displayed phenotypic brain abnormalities similar to that which has been noted in reports of AIDS patients upon tissue evaluation [65]. In an effort to circumvent the infectious step of HIV-1 completely, the entire HIV-1 genomic element is incorporated directly into the mouse genome, rendering expression in many tissues. And by excluding genes *gag* and *pol*, which are essential for virion production [66, 67], the model is rendered non-infectious [68]. This particular model in turn could be suitable for studying efficacy of therapeutic treatments and can be well suited for elucidating the underlying mechanisms contributing to HIV- associated disorders such as HAND, as well as reducing any hazards that are associated with an infectious model. Although there is no viral replication in these transgenic animals, viral proteins are continually expressed, stimulating the immune system in a manner comparable to HIV-1-infected individuals on cART [69]. HIV-1 transgenic rats that contain the HIV-1 genome have been shown to exhibit altered BBB integrity and increased monocyte infiltration into the brain [63, 70, 71]. In addition, HIV-1 protein mRNA has shown to be detectable in the brain of HIV-1 transgenic rats as young as 2 months of age and increases or decreases with age, depending on the specific brain area [72], in a pattern consistent with autopsy results in humans [73]. The development of transgenic mice creates a platform that serves a wealth of information but it has its limitations such as their inability to recapitulate a robust viral replication, and protein expression is induced by an antibiotic, which can be of concern [74]. More importantly, transgenic mice models, while advantageous, the scope in which they can be used is minimized. CNS disorders are an aggregate result of multiple pro-inflammatory mediators of viral as well as cellular origin which cannot be recapitulated when a single viral protein is expressed. HIV pathogenesis is a complexity of viral and host factors as well as host responses that together, causes inflammatory disorders. Therefore, animal models that mirrors HIV manifestations in humans as closely as possible are the models that will afford one to explore CNS disorders in its true capacity. With this need, a second type of mouse model was developed, the humanized mouse model in which immunocompromised mice were developed by numerous genetic deletions, including, NOD/scid-IL-2Rγ_c_^null^ (NSG), BALB/c-Rag2−/−γ_c_^−/−^, and NOD/scid (BLT) mice have been engrafted with human tissues to reconstitute the human immune system. Mice with a humanized immune system can sustain long-term chronic HIV replication and are susceptible to infection through the natural routes by which humans are exposed to the virus [61]. Unlike the transgenic mice expressing Tat/gp120, humanized mice incorporates HIV-1 infection of physiologically relevant targets. Thus, these mice exhibit a robust and systemic viral replication. In a recent report, mice permanently reconstituted with a human immune system and subsequently intraperitoneally injected with HIV-1 were able to reflect the course of viral infection as reported in humans and recapitulate similar HIV-associated neuropathological disorders. Specifically, these mice exhibited CD8^+^ T-cell depletion, development of meningitis and as infection progressed, entry of monocytes into the brain also accelerated [75]; phenomenons all which have been reported in HIV-patients [76–78]. Follow-up work by the same group report in HIV-1-positive humanized mice (infected with HIV at birth), as the infection progressed, neuronal integrity lessened, thereby revealing a correlation between continual viral replication and neuronal dysfunction [79]. HIV-1 infection not only affects the brain, it also affects multiple organs including the lungs [80]. Interestingly, the lung and brain share a common structural feature which are tight junction complexes consisting of transmembrane proteins, claudins, that maintain intercellular barriers [8, 14, 28, 81, 82]. Li *et al* report HIV-1 induces interstitial pneumonitis (IP), a serious complication of HIV-1 infection, in humanized mice by increasing pulmonary macrophage infiltration and decreasing expression of tight junction protein claudin-5 [83]. Although tight junctions occur in both endothelial and simple epithelial cells, claudin-5 is endothelial cell-specific of tight junctions [81]. Moreover, as mentioned previously, claudin-5 is a major determinant of tight junction proteins and decreased expression of claudin-5 has been observed in post-mortem tissues of HIV-1 patients [84], HIV and Tat-induced *in vitro* models [53] as well as in our current study of EcoHIV model. Studying HIV-1 pathogenesis, a species-specific infection of the human immune system, in totality was made possible by re-creating a human immune system in rodents. However, working with these mice and wild-type HIV-1 in the laboratory is hazardous. Additionally, due to their immunocompromised state, it is unclear if these mice are able to survive long enough to develop the mild to severe stages of neurocognitive disorders. The allogeneic transplant of human cells into these mice can mount a host immune response against itself that cannot be prevented. This graft-vs-host rejection is an additional caveat of humanized mice [85]. Lastly, another disadvantage of humanized mice is a significant variability between mouse batches. This is due to the highly variable nature of human immune cells from person to person [86]. Signifying the need for a more user-friendly, cost effective model to study HIV pathogenesis. In the current study, we utilized a murine-tropic chimeric virus, a derivative of HIV-1 in such a manner that the two genomes are identical with the exception of HIV-1 gp120 is replaced with MuLV gp80 [29–31], to model neuroinflammation in order to determine the role of activated platelets in mediating BBB dysfunction.

The BBB is a neurovascular unit composed of brain microvascular endothelial cells (BMECs), astrocytes, pericytes, perivascular microglia, and basal lamina [87]. Tight junctions, composed of proteins such as claudin-5 and occludin, form a restrictive barrier that regulates the transport of macromolecules and inflammatory cells into the brain and mediates BMEC-BMEC interaction, ultimately ensuring proper BBB integrity [8, 88]. It has been previously shown that HIV-1 viral proteins can alter tight junction expression resulting in increased BBB permeability [89, 90]. Using the EcoHIV infection model, we demonstrate that as early as two-weeks post-infection, BBB permeability is increased. This is thought to be mediated by the decreased expression of a key tight junction protein, claudin-5. This decrease was also observed in brain sections collected from infected mice two months post infection that was analyzed for claudin-5 immunoreactivity. This was not surprising since previous reports have shown that endothelial cells of microvessels have decreased tight junction protein expression [53]. Interestingly, we also observed a decrease in claudin-5 expression in macrovessels after EcoHIV infection, which may be a result of the broader tropism mediated by the pseudotyped virus and gp80 expression. However, in contrast, the expression of the tight junction protein, occludin, did not decrease. It is known from post-mortem tissues collected from HIVE patients that tight junction protein expression is variable, which may account for the differences observed between claudin-5 and occludin expression [15].

Platelets are now known to be involved in the immune response in addition to its commonly known functions in hemostasis. Therefore, it is not surprising that these cells and their subsequent activation have been implicated in several inflammatory disorders resulting from infection. For example, low platelet number or thrombocytopenia is observed and implicated as playing a role in altering the integrity of the BBB in cerebral malaria infections [91]. Thrombocytopenia has also been consistently identified in individuals with dengue virus infection [92]. In these patients, vascular leakage of proteins/hemorrhaging and clinical demise has been correlated with platelet levels [93]. Individuals infected with dengue virus that exhibit low platelet number have a significantly higher risk of developing neurological complications as compared to patients with normal platelet behavior [94]. Thrombocytopenia has also been associated with HIV disease progression [95, 96]; low platelet counts were determined as a clinical sign of AIDS before HIV was identified as the causative agent [97]. This decline in platelet number arises through multiple mechanisms including increased platelet activation and clearance [98, 99], in addition to platelets forming complexes with activated CD4^+^ T-cells and monocytes resulting in respective aggregates, which may mask accurate platelet counts [100]. Further, a dysfunction in platelet production may also explain the thrombocytopenia that often occurs with HIV infection [101]. HIV-associated thrombocytopenia is also caused by HIV antibodies cross-reacting with platelet glycoproteins [102]. Several studies have shown cross-reactivity of HIV-1 glycoprotein gp41 with platelet gpIIb/IIIa in some cases of idiopathic thrombocytopenic purpura (ITP) in HIV-infected patients [103, 104]. This molecular mimicry between HIV and platelet antigens contributes directly to platelet destruction/decline. Platelets are activated in HIV-1 patients [43, 98] and while platelets are important for homeostasis [105, 106], they also serve an important role in immune responses by releasing certain molecules such as CD40L [56, 107–109]. CD40L has been well studied for its role in regulating B-cell response; specifically, B-cell activity (proliferation, differentiation, isotype switching, memory B-cell generation, and germinal center formation) is dependent upon the binding of CD40L to its receptor, CD40, on B-cells [110, 111]. 95% of circulating CD40L is derived from platelets [50] and platelet activation is increased in HIV patients; similarly, concentrations of CD40L are also increased [37, 112]. Recently, reports have shown that platelet-derived CD40L stimulates B-cell activity by enhancing Ig production [113] and these circulating, stimulated B-cells are excreting anti-gpIIb/IIIa antibodies in ITP patients at increased levels [114]. Therefore, in HIV patients, dysregulation of B-cell development is another factor that induces thrombocytopenia as the result of an increased stimulatory environment

Although we have previously reported that sCD40L mediates HIV-1 Tat-induced BBB permeability [37], this study was limited in analyzing the effect of a single viral protein as compared to full-length infection. We have conveyed here that EcoHIV infection induces platelet activation as evident by increased concentrations of platelet releasates, sCD40L and PF4, and a significant decrease in bleeding time. Further, we also demonstrate the importance of CD40L signaling in BBB dysfunctions, as BBB permeability was comparable in CD40L knock-out mice infected with EcoHIV to that of uninfected mice. Although there was no significant change in BBB permeability in these knock-out mice, platelets were indeed activated, as evident by increased platelet activation markers as well as decreased platelet counts, indicating platelet clearance. This data further supports the notion that sCD40L influences HAND pathogenesis by inducing BBB dysfunction, ultimately resulting in the invasion of proinflammatory leukocytes into the brain and the establishment of a neurotoxic environment.

Current treatment of cART alone is insufficient to abrogate the pathogenesis of HAND, thus signifying the need to develop adjunctive therapy that can be used in conjunction with cART. Targeting the CD40/CD40L signaling axis is a suitable approach to alleviate the neuropathies associated with HIV-1 infection, however, consideration must be given to the fact that CD40/CD40L signaling is crucial for the costimulatory signaling for B cell development and function, as well as the development of T cell dependent immune responses [115]. Considering that 95% of sCD40L is derived from activated platelets [50], the dampening of platelet activation and subsequently reducing plasma sCD40L levels, can be promising considering surface expressed CD40L on other cell types, such as B and T cells, would not be affected. Reducing sCD40L levels by attenuating platelet activation would still allow for the needed humoral and adaptive immune responses within HIV-1 infected individuals, thus making antiplatelet therapy an attractive adjunct to cART.

We explored this avenue of reducing platelet activation by administering eptifibatide, an antiplatelet drug and an integrin gpIIβ/IIIα (fibrinogen receptor) antagonist that is clinically used to treat patients with thromboembolisms and ACS [55, 57, 58], to EcoHIV infected mice. Fibrinogen receptors on platelets are important for proper platelet aggregation, yielding further platelet activation, degranulation, and release of stored mediators [116]. Reports have demonstrated that eptifibatide regulates platelet surface expression of P-selectin [107] and decreases aggregation thereby reducing lung inflammation induced by influenza A infection [117]. We demonstrate here that one-week of eptifibatide treatment in EcoHIV-infected mice one month post infection, significantly reduced BBB permeability and the associated platelet activation and sC40L levels via normalization of claudin-5 and ZO-1expression. However, when the treatments were aborted and infected mice were sacrificed one month later (for a total time period of 2 months post infection), we observed an increase in CD62P^+^ expression comparable to that of mice treated with saline, indicating that platelet activation is no longer blocked. Ultimately, this report strengthens the significance of consistent antiplatelet therapy adjunctive to cART, which may prove to be a powerful and beneficial way of reducing the prevalence of HAND.

In conclusion, these results validate the EcoHIV infection mouse model as an ideal tool for defining pathopsychological mechanisms underlying neurinflammatory responses during HIV infection.

## Supporting Information

S1 Fig S100B concentrations are increased in EcoHIV-infected mice. (A) At two mpi, plasma from EcoHIV-infected mice and uninfected controls were analyzed for levels of S100B via ELISA, which was significantly higher, compared to uninfected mice (n = 3 for each group). (B) One mpi, EcoHIV infected mice treated with eptifibatide showed lower levels of plasma S100B expression compared to saline treated mice (n = 3).
(TIF)

S2 Fig EcoHIV infection alters the expression of tight junction proteins and adhesion molecules. **(A)** Brain tissues from uninfected and EcoHIV-infected mice (two mpi) were homogenized, protein lysates were collected, and probed for ZO-1 expression via western blot. A representative blot image shows a decrease in ZO-1 expression in EcoHIV infected mice. Quantification of ZO-1 protein levels from uninfected (n = 3) and EcoHIV infected (n = 3) mice confirms the decreased expression. **(B)** qRT-PCR analysis of ZO-1 expression in brain tissues harvested from both infected WT mice and uninfected controls showed a decrease of ZO-1 transcripts in infected mice (n = 3 for each group). qRT-PCR analysis of **(C)** I-CAM-1 expression and **(D)** V-CAM-1 expression in brain tissues harvested from both infected WT mice and uninfected controls showed increased transcripts in infected mice (n = 3 for each group) and **(E)** Occludin transcripts in infected mice and uninfected control mice (n = 3 for each group) reman unaltered.
(TIF)

S3 Fig EcoHIV infection alters the expression of ZO-1 and I-CAM-1 in a CD40L dependent manner. **(A)** qRT-PCR analysis of ZO-1 expression in brain tissues harvested from both infected and uninfected WT and CD40L KO mice showed a reduction of ZO-1 transcripts in infected WT mice as compared to uninfected WT conrols, but no difference was found in the CD40L KO counterparts (both groups n = 3). **(B)** qRT-PCR analysis of I-CAM-1 expression in brain tissues harvested from both infected and uninfected WT and CD40L KO mice showed an increase of I-CAM-1 transcripts in infected WT mice as compared to uninfected WT controls, but not in the CD40L KO counterparts (both groups n = 3).
(TIF)

S4 Fig Eptifibatide treatment restores I-CAM-1 and ZO-1 expression in EcoHIV-infected mice. **(A)** Expression of I-CAM-1 (n = 4) and **(B)** ZO-1 (n = 3) proteins in brain tissues of EcoHIV infected mice was analyzed by western blot. One mpi, mice treated with eptifibatide showed lower levels of I-CAM-1 expression compared to saline treated mice. Further, ZO-1 levels were significantly higher in eptifibatide treated mice as compared to saline controls. The corresponding densitometric quantitation is shown below the respective blot.
(TIF)
