# Supplementary figures and images for: Modeling HIV-1 Induced Neuroinflammation in Mice: Role of Platelets in Mediating Blood-Brain Barrier Dysfunction
Source: PLoS One. 2016 Mar 17;11(3):e0151702. doi: 10.1371/journal.pone.0151702 (PMC4795798; doi:10.1371/journal.pone.0151702)

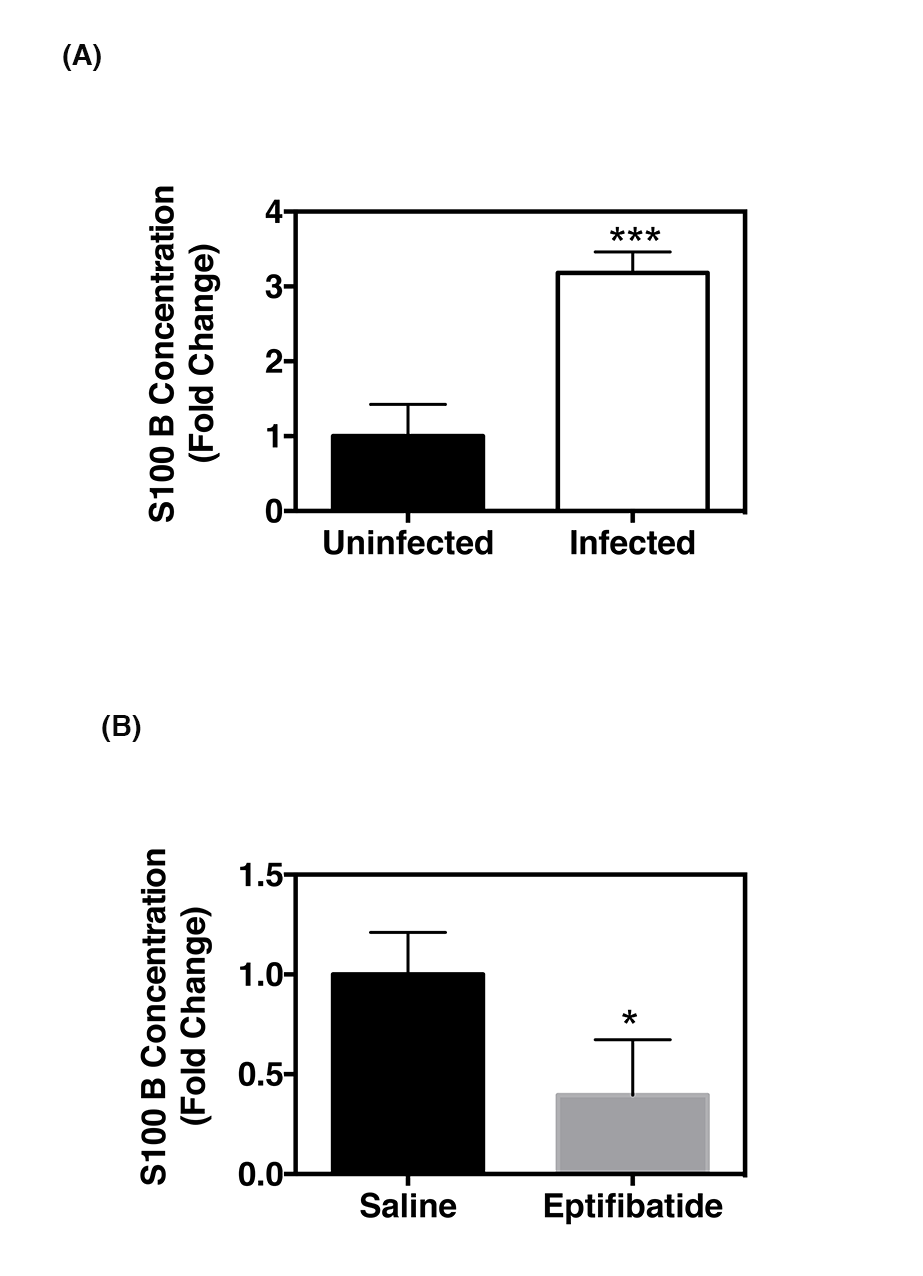

Supplement: S1 Fig — (A) At two mpi, plasma from EcoHIV-infected mice and uninfected controls were analyzed for levels of S100B via ELISA, which was significantly higher, compared to uninfected mice (n = 3 for each group). (B) One mpi, EcoHIV infected mice treated with eptifibatide showed lower levels of plasma S100B expression compared to saline treated mice (n = 3). (TIF) [file pone.0151702.s001.tif]

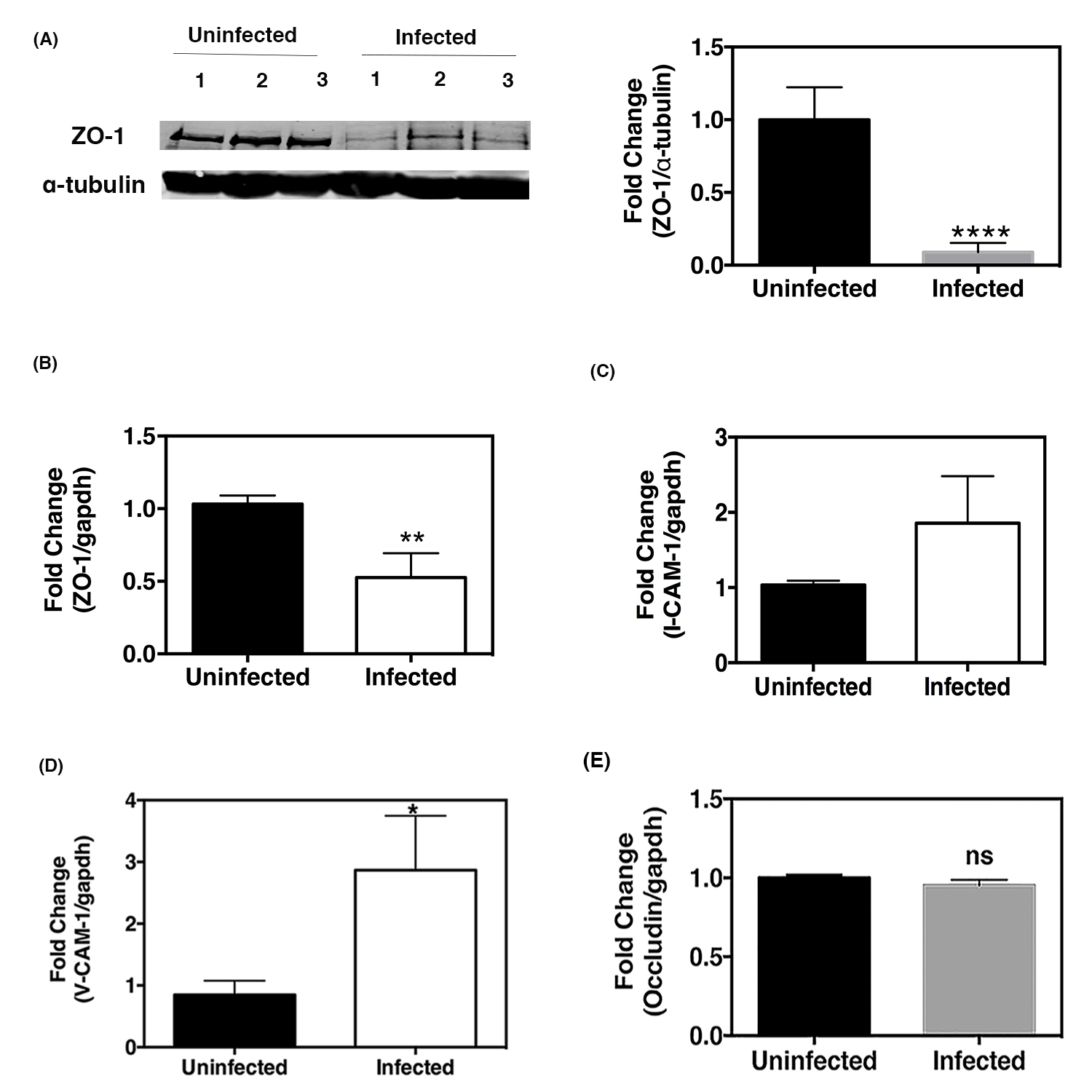

Supplement: S2 Fig — (A) Brain tissues from uninfected and EcoHIV-infected mice (two mpi) were homogenized, protein lysates were collected, and probed for ZO-1 expression via western blot. A representative blot image shows a decrease in ZO-1 expression in EcoHIV infected mice. Quantification of ZO-1 protein levels from uninfected (n = 3) and EcoHIV infected (n = 3) mice confirms the decreased expression. (B) qRT-PCR analysis of ZO-1 expression in brain tissues harvested from both infected WT mice and uninfected controls showed a decrease of ZO-1 transcripts in infected mice (n = 3 for each group). qRT-PCR analysis of (C) I-CAM-1 expression and (D) V-CAM-1 expression in brain tissues harvested from both infected WT mice and uninfected controls showed increased transcripts in infected mice (n = 3 for each group) and (E) Occludin transcripts in infected mice and uninfected control mice (n = 3 for each group) reman unaltered. (TIF) [file pone.0151702.s002.tif]

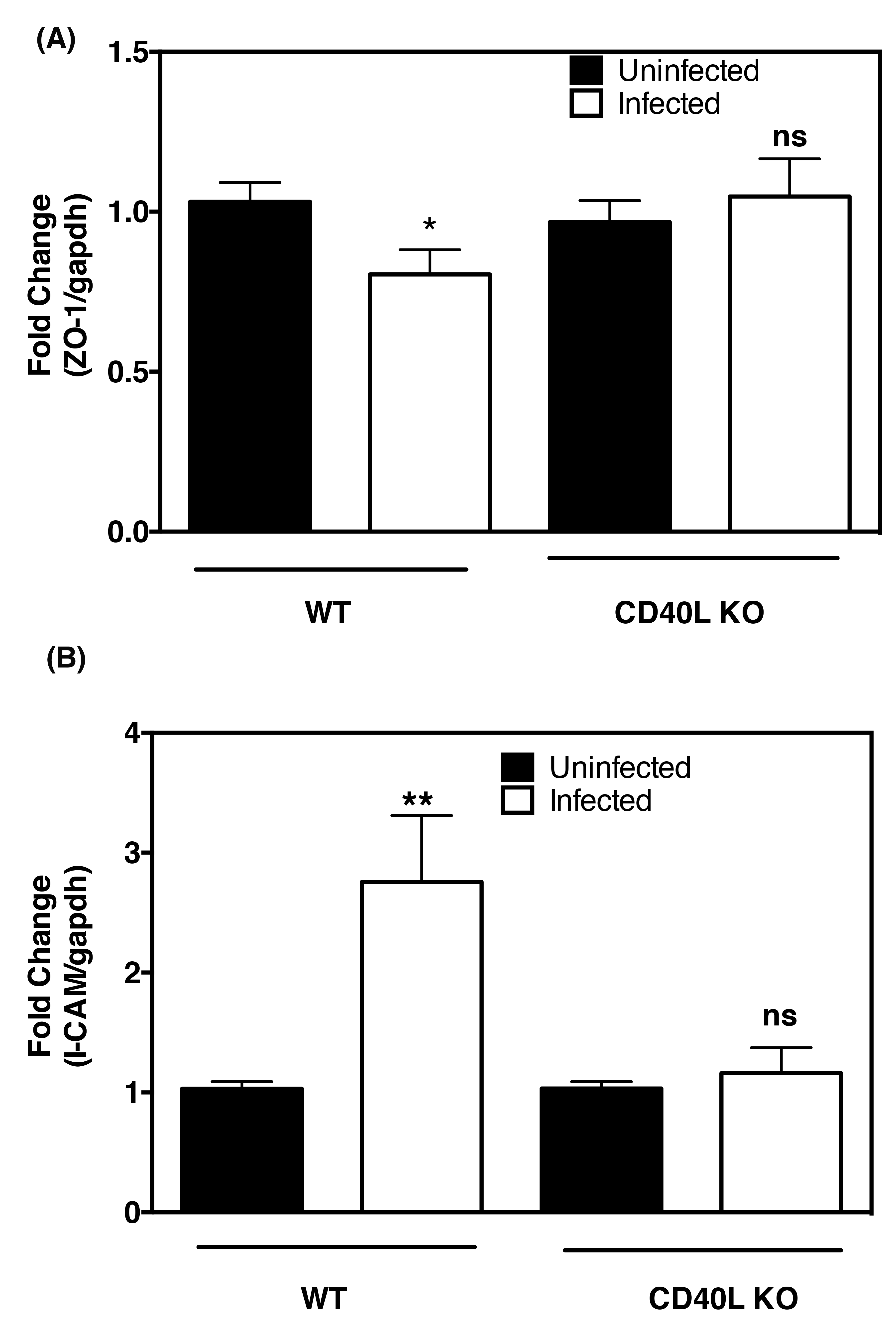

Supplement: S3 Fig — (A) qRT-PCR analysis of ZO-1 expression in brain tissues harvested from both infected and uninfected WT and CD40L KO mice showed a reduction of ZO-1 transcripts in infected WT mice as compared to uninfected WT conrols, but no difference was found in the CD40L KO counterparts (both groups n = 3). (B) qRT-PCR analysis of I-CAM-1 expression in brain tissues harvested from both infected and uninfected WT and CD40L KO mice showed an increase of I-CAM-1 transcripts in infected WT mice as compared to uninfected WT controls, but not in the CD40L KO counterparts (both groups n = 3). (TIF) [file pone.0151702.s003.tif]

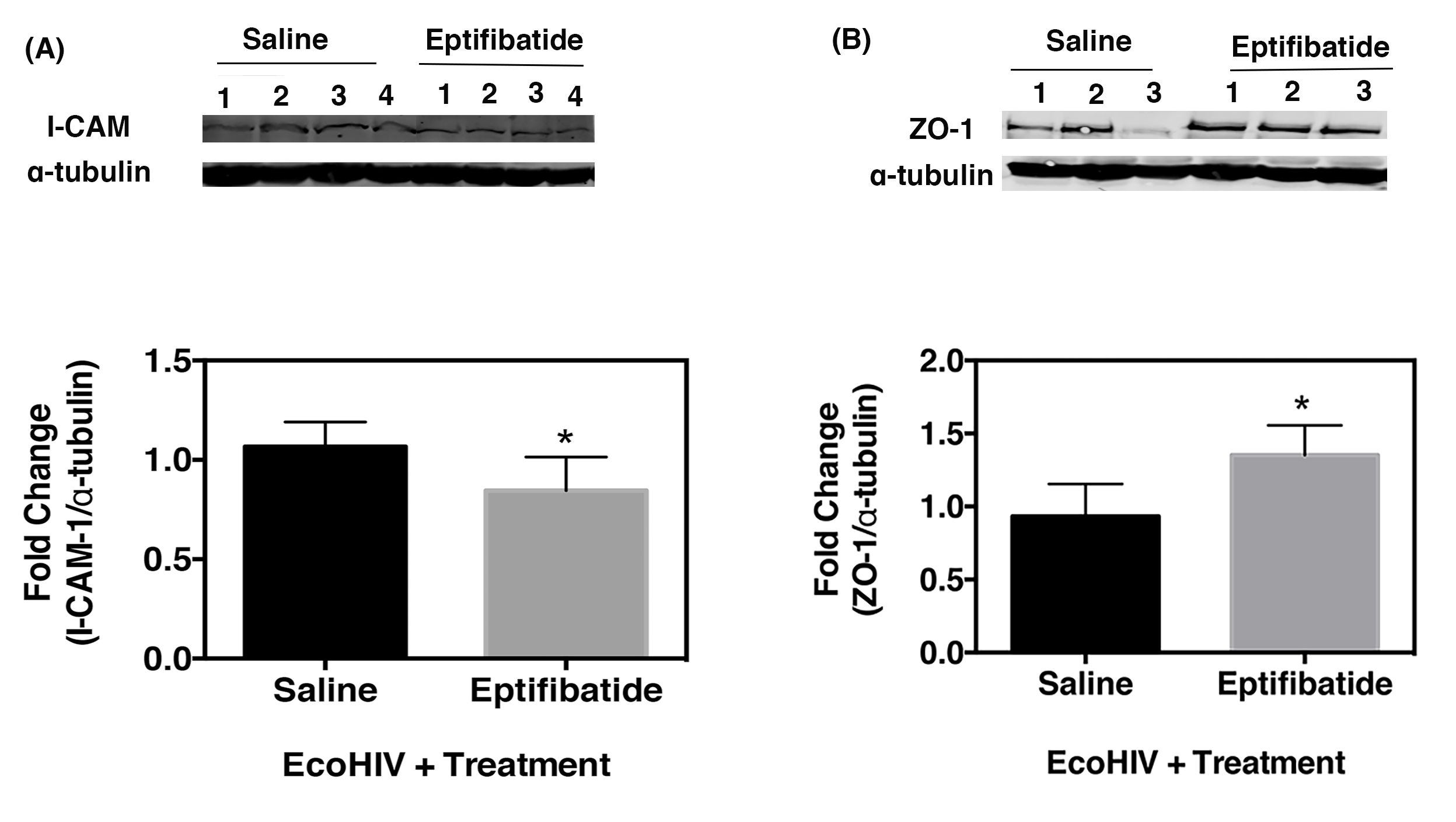

Supplement: S4 Fig — (A) Expression of I-CAM-1 (n = 4) and (B) ZO-1 (n = 3) proteins in brain tissues of EcoHIV infected mice was analyzed by western blot. One mpi, mice treated with eptifibatide showed lower levels of I-CAM-1 expression compared to saline treated mice. Further, ZO-1 levels were significantly higher in eptifibatide treated mice as compared to saline controls. The corresponding densitometric quantitation is shown below the respective blot. (TIF) [file pone.0151702.s004.tif]
